# Dihydrotestosterone increases the risk of bladder cancer in men

**DOI:** 10.1007/s13577-019-00255-3

**Published:** 2019-05-22

**Authors:** Dorota Gil, Marta Zarzycka, Joanna Dulińska-Litewka, Dorota Ciołczyk-Wierzbicka, Małgorzata Lekka, Piotr Laidler

**Affiliations:** 1 Chair of Medical Biochemistry, Jagiellonian University Medical College, Kraków, Poland, ul.Kopernika 7, 31-034 Kraków, Poland; 2 Laboratory of Biophysical Microstudies, The Henryk Niewodniczański Institute of Nuclear Physics, Polish Academy of Sciences, Poland, ul. Radzikowskiego 152, 31-342 Kraków, Poland

**Keywords:** Bladder cancer, Androgen receptor, Akt, GSK-3β, eIF4E

## Abstract

Men are at a higher risk of developing bladder cancer than women. Although the urinary bladder is not regarded as an sex organ, it has the potential to respond to androgen signals. The mechanisms responsible for the gender differences remain unexplained. Androgen receptor (AR) after binding with 5α-dihydrotestosteron (DHT) undergoes a conformational change and translocates to nucleus to induce transcriptional regulation of target genes. However androgen/AR signaling can also be activated by interacting with several signaling molecules and exert its non-genomic function. The aim of present study was to explain whether the progression of bladder cancer in men is dependent on androgen/AR signaling. Studies were carried out on human bladder cancer cell lines: HCV29, T24, HT1376 and HTB9. Bladder cancer cells were treated for 48 h with 10 nM DHT or not, with replacement after 24 h. Expression of cell signaling proteins, was analyzed using Western Blot and RT-PCR. Subcellular localization of protein was studied using the ProteoExtract Subcellular Proteome Extraction Kit and Western blot analysis. We showed that DHT treatment significantly increased AR expression in bladder cell line HCV29. We also observed DHT-mediated activation of Akt/GSK-3β signaling pathway which plays a central role in cancer progression. Presented results also show that androgen/AR signaling is implicated in phosphorylation of eIF4E which can promote epithelial–mesenchymal transition (EMT). We indicate that AR plays an essential role in bladder cancer progression in male patients. Therefore, androgen-activated AR signaling is an attractive regulatory target for the inhibition or prevention of bladder cancer incidence in men.

## Introduction

Bladder cancer (BC) is currently the one of the very widespread genitourinary tract malignancy, the fifth most common cancer in men and the nineteenth in women [1, 2]. Different environmental and lifestyle factors, such as industrial chemicals or cigarette smoking, have been believed as the reasons for sex-related differences. Although the urinary bladder is not regarded as an sex organ, it has the potential to respond to androgen signals, because is derived from the endoderm of the urogenital sinus which expresses the AR [3]. AR signaling was shown as the crucial oncogenic driver of prostate cancer, it is also significantly associated with tumor progression in other solid tumors, such as lung, kidney, breast and bladder cancers [4, 5]. The mechanisms responsible for the gender differences remain unclear but AR signaling pathway has been proposed as an important factor that is involved in the development and progression of BC. Studies in animal models evidence that AR in urothelium may play a crucial role in cancerogenesis and progression of BC [6]. Shiota et al. showed that men with prostate cancer who underwent androgen deprivation therapy had a lower risk of development of bladder cancer in comparison to those undergoing surgery or radiotherapy, only [7]. Also, Fahmy et al. described high incidence of prostatic adenocarcinoma in cystoprostatectomy specimens undergone for bladder urothelial carcinoma [8].

Androgen receptor in the cytoplasm occurs in the complex with heat shock proteins, and upon binding DHT, AR is released from heat shock proteins and translocates to nucleus to induce transcriptional regulation of target genes. This AR-signaling pathway is known as the genomic pathway. However, androgen/AR signaling can also be activated by an alternative mechanism independent on androgen binding that includes its phosphorylation by kinases [9, 10]. Studies on prostate cancers showed that binding of androgen to AR in the cytoplasm may initiate signal transduction pathways to regulate cellular proliferation and migration, known as non-genomic pathway, that requires neither AR nuclear translocation nor DNA binding [11–13]. In addition, non-genomic AR signaling may be mediated by a membrane-bound AR [14, 15]. Signaling molecules activated by AR in transcription-independent manner includes Src, Ras, MAPK, Akt, PKC, PLC, EGFR and other secondary messenger proteins [15]. It is possible that non-genomic activity influences AR genomic activity and that of other nuclear receptor.

Androgen receptor has been detected in human BC in a number of studies but it is not clear whether its expression level is important in bladder cancer progression [9, 10, 16, 17]. The data suggest that AR expression is more often upregulated in early stages of disease than in advanced stages of BC [17]. Because men have higher circulating level of androgen than females and AR can be variably expressed also in female tumors, probably that combination of AR expression level and elevated androgen signaling play the essential role in gender distinction characterizing BC.

The aim of present study was to elucidate the mechanism of androgen/AR signaling in promotion of bladder cancer in male patients.

## Materials and methods

### Cell culture

HCV29 (nonmalignant transitional epithelial cells of the ureter), T24 (transitional cancer cells of the urine bladder), HT1376 (urinary bladder carcinoma, grade III) and HTB9 (urinary bladder cancer grade II) cell lines were obtained from American Type Culture Collection. These cell lines were then purchased from ATCC by M. Lekka’s lab and authenticated by short tandem repeat analysis by LGC Standard. All cell lines are examined annually for mycoplasma infection by PCR method. The cells were cultured in RPMI-1640 medium supplemented with 10% fetal calf serum (FCS) (Gibco) and 1% penicillin/streptomycin [18].

### Cell culture treatment

Bladder cancer cells were treated for 48 h with DHT, 10 nM, (Sigma-Aldrich) with replacement after 24 h. Dihydrotestosterone was prepared at a stock concentration of 0.2 M in ethanol (Sigma-Aldrich). A dose of 10 nM in serum-free Opti-MEM (Gibco) was used as the physiological dose capable of effectively activation of AR expression. As the vehicle control, bladder cells were cultured with 0.1% ethanol. Since there were no differences between ethanol-treated cells (not shown) and untreated cells, the latter were selected as control cells in each experiment. Bladder cancer cells 24 h after seeding were treated for 0.5 h or 1 h with 10 nM DHT, 0.1% ethanol as a control in serum-free Opti-MEM and afterwards used to study non-nuclear AR signaling.

### Preparation of cytoplasmic, membrane, nuclear and cytoskeletal cell lysates

Cytoplasmic, membrane, nuclear and cytoskeletal extracts were prepared using the ProteoExtract^®^ Subcellular Proteome Extraction Kit (MERCK Millipore) by following the manufacturer’s instructions.

### Western blot analysis

Cells lysis and western blot were carried out as previously described [18]. Antibodies for: ILK, Akt, E-cadherin, Vimentin, GSK-3β, phospho-GSK-3β (Y216), β-catenin (all Transduction Laboratories BD), AR, Src, phospho-Src (Y416), phospho-ERK (T202/Y204), phospho-Akt (S473), phospho-GSK-3β (S9), D_1_, D_3_ and phospho-eIF4E (S209) (all Cell Signaling Technology Inc.), N-cadherin (R&D) and β-actin, ZEB1, (all Sigma) SNAIL (ABGENT), Calnexin, HSP-90 (all Calbiochem) were used to detect indicated proteins. The presence of the primary antibody was revealed with horseradish peroxidase-conjugated secondary antibodies diluted 1:2000 (Cell Signaling Technology Inc) and visualized with an enhanced chemiluminescence detection system (Bio-Rad) as previously described [19]. β-Actin served as a loading control. All immunoblots were stripped with stripping buffer containing 25 mM glycine–HCl, pH 2, 1% (wt/v) SDS for 30 min, and incubated in antibody against β-actin (dilution, 1:3000; Sigma-Aldrich), which served as a loading control. To obtain quantitative results, immunoblots were scanned using the public domain ImageJ software (National Institute of Health). Each data point was normalized against its corresponding actin data point.

### In vitro wound healing/migration assay

The in vitro model of wound healing was used to compare the migration and proliferation potential of bladder cancer cell lines in presence or absence of DHT. For wound healing assay, cells were grown in RPMI-1640 medium supplemented with 10% fetal calf serum in 24-well plates until confluent. A small linear scratch was created in the confluent monolayer by gently scraping with sterile 1 ml pipette tip. The cells were washed with medium to remove cellular debris and serum-free Opti-MEM media with 10 nM DHT or 0.01% ethanol as control were added. Twenty-four hours later, images of the migrated cells were taken using digital camera (Nikon), connected to the inverted microscope. The assay was repeated thrice in duplicate. Wound area was calculated by manually tracing the cell-free area in captured images using the public domain ImageJ software. The migration rate was expressed as the percentage of wound closure.

### RNA extraction, cDNA synthesis and RT-PCR analysis

RNA extraction, preparation of cDNA and RT-PCR reaction were carried out as previously described [18]. The following specific primers were used:

GAPDH; (59 °C, 30 s): 5′CACCGCCTCGGCTTGTCACAT 3′ and 5′CTGCTGTCTTGGGTGCATTGC3′;

N-CADHERIN; (60 °C, 40 s): 5′GTGCCATTAGCCAAGGGAATTCAGC3′ and 5′CGAGGATACTCACCTTGTCCTTGCG 3′;

E-CADHERIN; (60 °C, 40 s): 5′GCCAAGCAGCAGTACATTCTACACG3′ and 5′GTCGTTCTTCACGTGCTCAAAATCC3′;

AR; (58, 30 s): 5′ TGTCAACTCCAGGATGCTCTACTT3′ and 5′ATTCGGACACACTGGCTGTACA3′

The PCR reaction products were separated electrophoretically on 2% agarose gel and visualized with ethidium bromide.

### Statistical analysis

Each variable was tested using the Shapiro–Wilk *W* test for normality. Homogeneity of variance was assessed with Levene’s test. Since the distribution of the variables was normal and the values were homogeneous in variance, all statistical analyses were performed using one-way analysis of variance (ANOVA) followed by Dunnett’s post hoc comparison test to determine which values differed significantly from controls. The analysis was made using Statistica software (StatSoft). Data were presented as mean ± SD. Data were considered statistically significant at p* < 0.05, p** < 0.01, p*** < 0.001.

## Results

### Expression of AR in human bladder and cancer cell lines

**Fig. 1:**
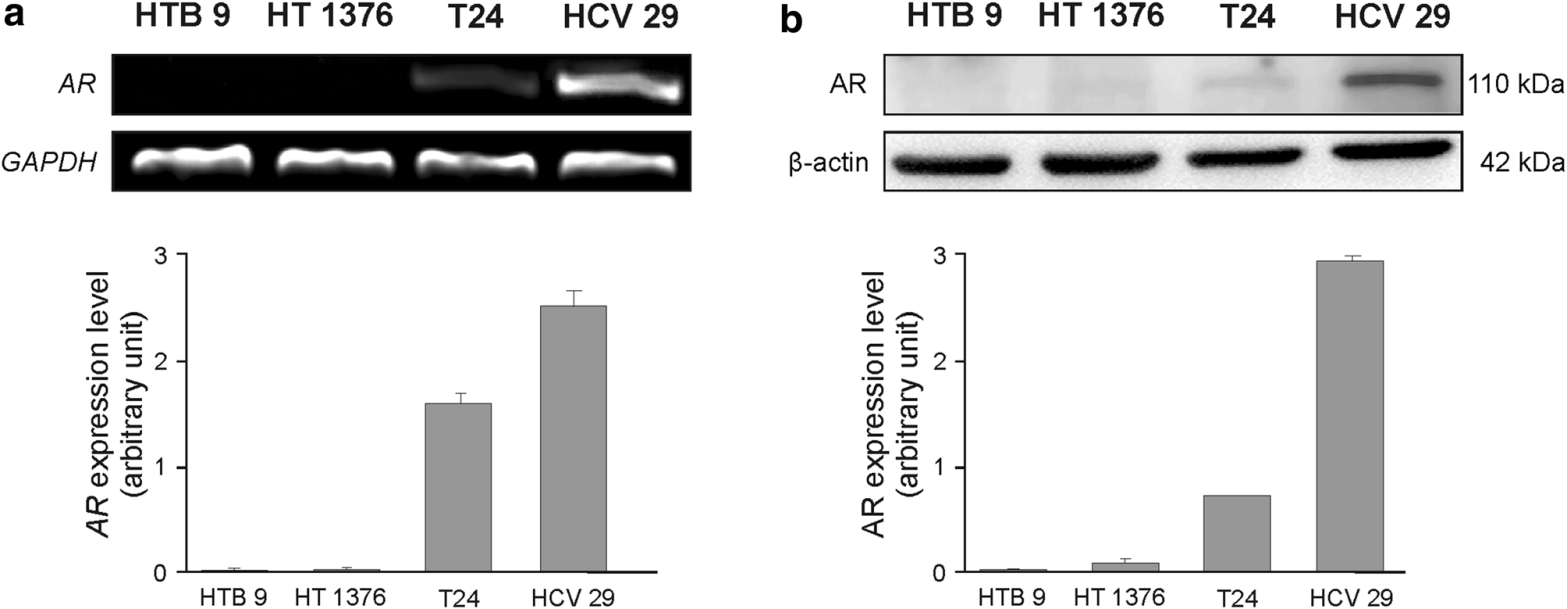
Expression of AR in HTB 9, HT1376, T24 and HCV29 bladder cell lines after 48 h treatment with 10 nM DHT. **a** AR expression on mRNA level was determined by reverse transcription-PCR. As an intrinsic control, the GAPDH mRNA level was measured in the samples. The relative level of each mRNA normalized against its corresponding GAPDH and expressed as relative optical density. Data obtained from three separate analyses are expressed as mean ± SD. **b** Protein expression was analyzed by Western blot. Total protein loading was determined by probing the membranes for β-actin. The relative level of each protein normalized against its corresponding β-actin and expressed as relative optical density. Data obtained from three separate analyses are expressed as mean ± SD

Urinary bladder cancer cell lines are very important research tools for studying biological and molecular mechanisms in cancer progression. Cell lines T24, HTB9 and HT1376 are described as representative models for study of human urinary bladder carcinogenesis because those lines cover the more frequent subtypes of this kind of tumor, representing a less aggressive phenotype and the later bearing more invasive and metastatic properties [20, 21]. Additionally, HCV29 as nonmalignant transitional epithelial cells of the ureter is a commonly treated as right reference, non-cancerous cell line in this model. We investigated the expression of androgen receptor in human bladder cancer cell lines T24, HTB9, and HT1376 as well as a nonmalignant transitional epithelial cells of the ureter HCV29 by Western blotting (Fig. 1).

AR expression was found to be the strongest in the transitional epithelial cells HCV29 and in transitional cell carcinoma T24. Both cell lines have been derived from men as well as more aggressive cell line HTB9 from grade II. The expression of AR was not found in HTB9 and in cell line HT1376 from grade III carcinoma, which is of female origin. These results suggested that AR expression is higher in non-cancer stage and in the early stages of bladder cancer progression, and likely decreases as the disease progresses.

### Effects of DHT on androgen receptor expression in bladder cell lines

**Fig. 2:**
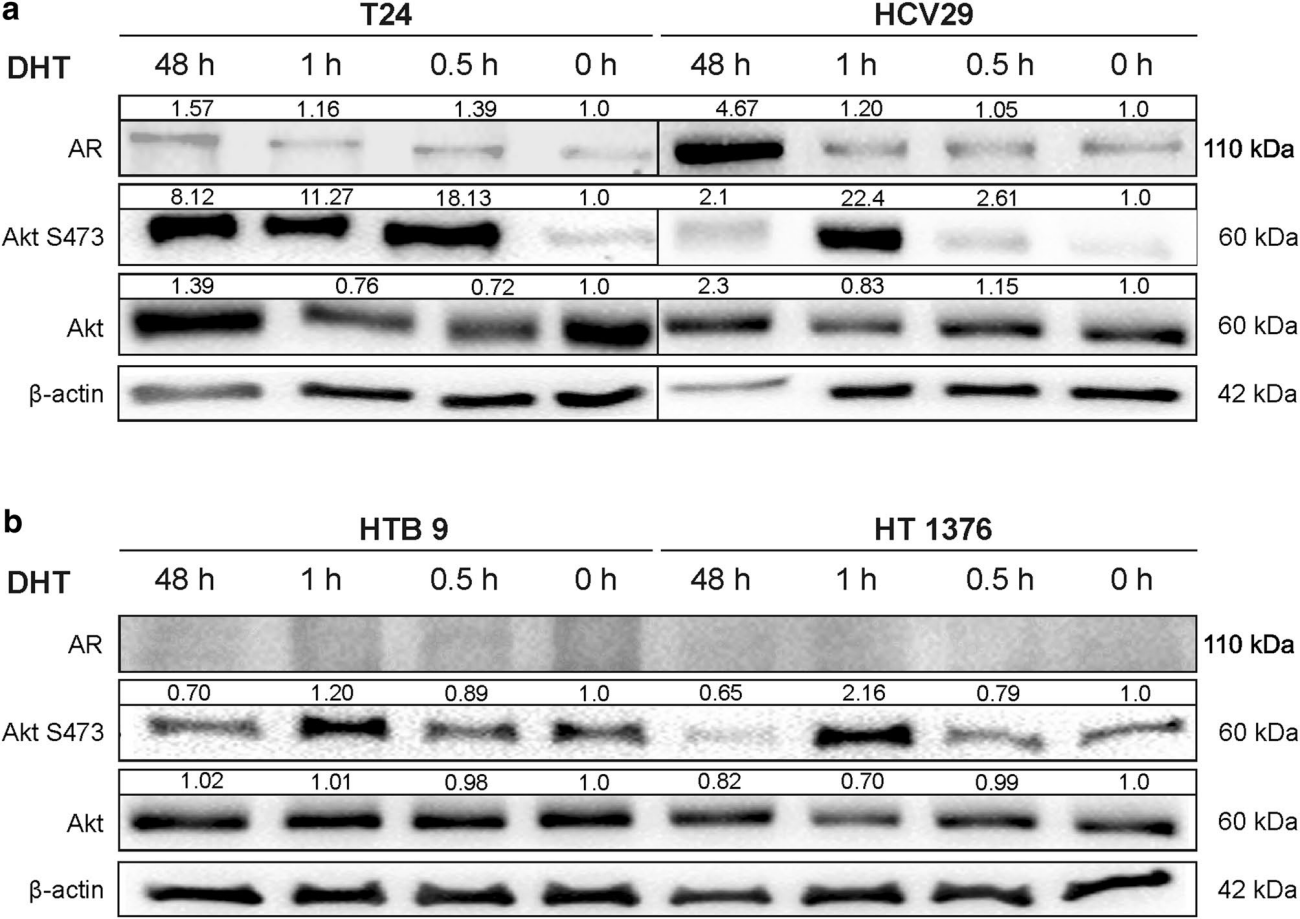
The effect of androgen (10 nM DHT) treatment of bladder cells on androgen receptor expression and Akt activation in non-genomic action of AR in 0.5 h; 1 h and 48 h incubation. Densitometry was used to normalize AR to β-actin protein level, and phosphorylated Akt to total Akt and to β-actin protein level. Presented are representative membranes of at least three independent experiments with similar results

**Fig. 3:**
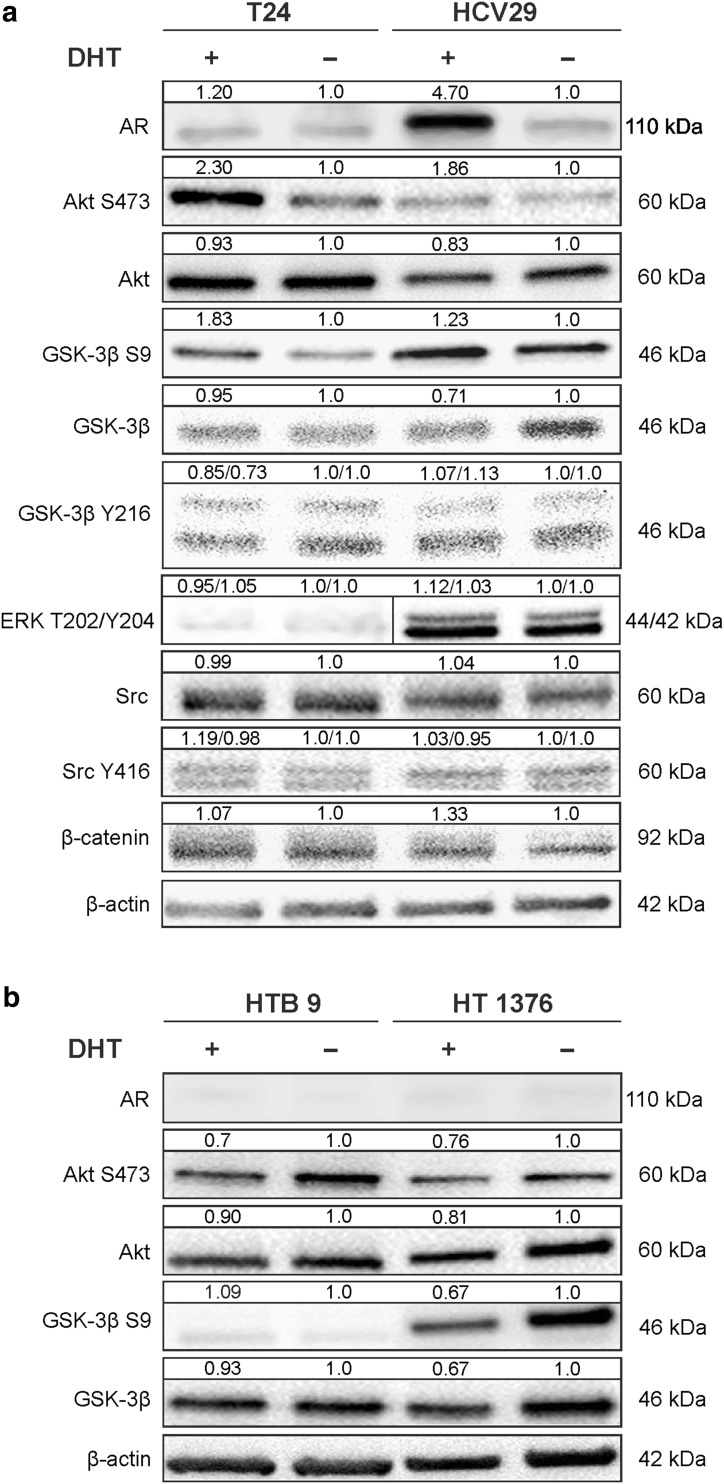
The effect of androgen (10 nM DHT) treatment of bladder cells on androgen receptor expression and action of AR on downstream signaling target proteins after 48 h incubation. **a** The effect of DHT treatment of transitional cells: HCV29 and T24. **b** The effect of DHT treatment of aggressive bladder cell lines HTB9 and HT1376. Densitometry was used to normalize to β-actin protein level and for quantitative comparison after DHT treatment. Presented are representative membranes of at least three independent experiments with similar results

We assessed changes in androgen receptor expression on protein level following 10 nM DHT treatment in bladder cell lines in comparison to control condition by Western blot. As shown Figs. 2 and 3, DHT treatment for 48 h increased significantly (4.7-fold) AR expression in nonmalignant transitional epithelial cells of the urether HCV29. In bladder cancer cell lines AR expression was marginally upregulated (1.2-fold) only in transitional cancer cell line T24 (Figs. 2, 3). These results suggest that androgen can directly stimulate the expression of its own receptor, especially at the early stages of cancer initiation.

### Non-nuclear AR signaling

**Fig. 4:**
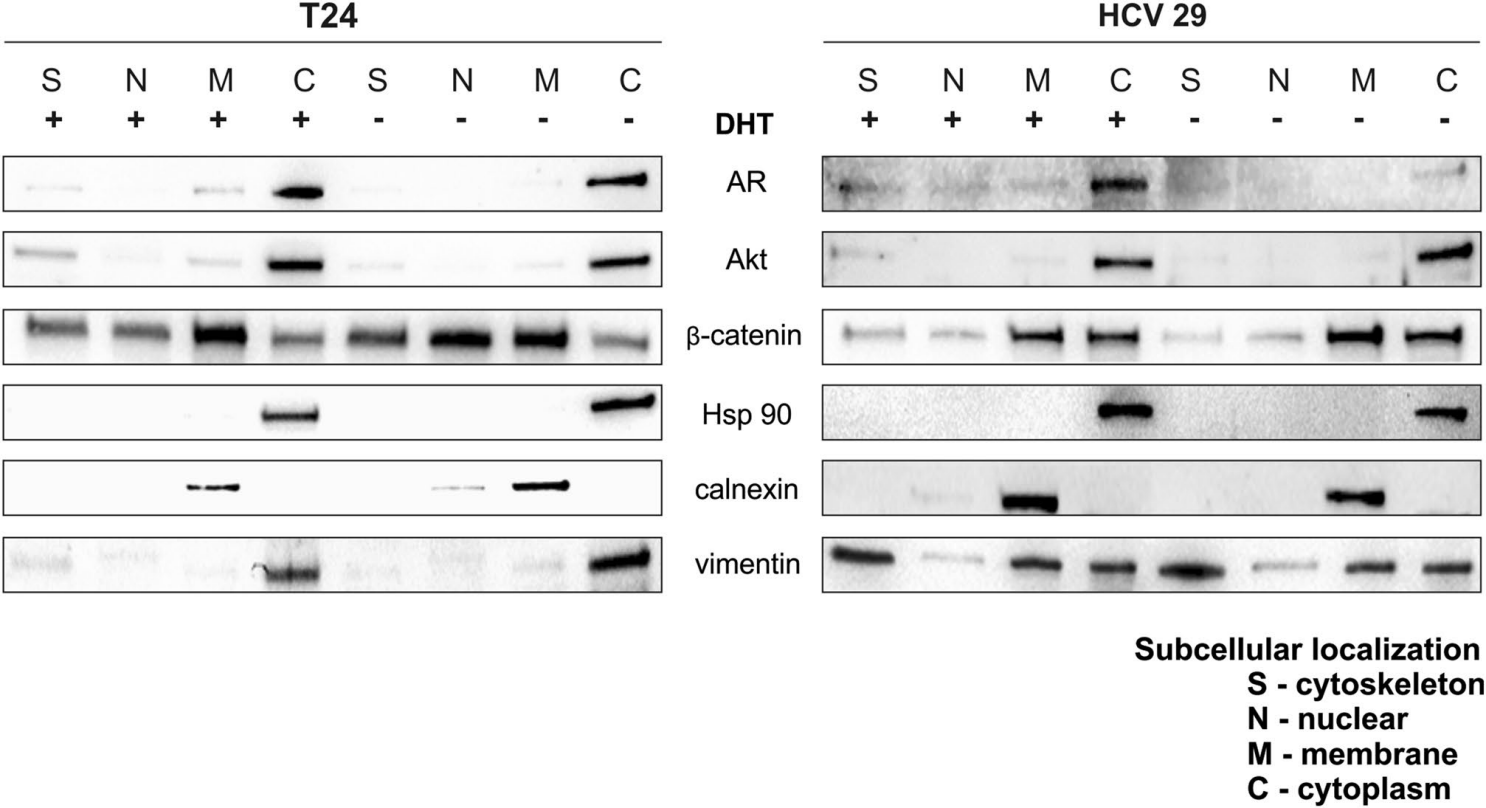
Androgen**-**dependent cellular localization of AR, Akt or β-catenin. Cytoplasmic (C), nuclear (N), membrane (M) and cytoskeleton (S) proteins were extracted as described in Materials and Methods. Calnexin, HSP 90 and vimentin were used as marker proteins specific for the appropriate fraction. Data were obtained from triplicate experiments

Androgen receptor non-genomic action originates in the cytoplasm or at the plasma membrane. Signaling molecules activated by AR include Src family, MAPK, PKC, Akt and other second messenger proteins. We examined whether androgen stimulation led to expression and activation of Src, Erk and Akt signaling pathways. Expression of activated molecules was monitored by phospho-specific antibodies. The increase of Akt phosphorylation was visible after 0.5 h treatment with DHT in both cell lines. The highest increase was observed after 0.5 h for T24 and after 1 h for HCV 29 and was still high after 48 h compared to the control. A rapid response after 1 h DHT treatment, was, however, detected also in cell lines HTB9 and HT1376, which did not express the classical AR, but in longer time 48 h, the effect was reduced in comparison to control cells (Fig. 2). The results indicated that androgen/AR interaction activates significantly Akt throughout the increase (2.3-fold) of its phosphorylation on Ser 473 at early stage of cancer cell line T24 and in nonmalignant transitional epithelial cell line HCV29 (1.86-fold) with slight difference in total Akt expression after 48 h (Fig. 3a). Upon activation, Akt phosphorylate a lot of other proteins including glycogen synthetase kinase 3 (GSK3). GSK-3β kinase activity is inhibited by direct phosphorylation at Ser 9 by Akt. DHT supplementation was sufficient for meaningful increase of phosphorylation of Ser 9 GSK-3β. Because the key proteins regulated by GSK-3β are β-catenin, we next examined the relationship between the higher phosphorylation of Ser 9 GSK-3β and the levels of total β-catenin and its nuclear accumulation. We detected increased total levels of β-catenin only in HCV29 cell line (Fig. 3a) but we did not observe any differences in nuclear translocation of β-catenin after DHT treatment in both tested cell lines (Fig. 4). We did not notice activation of the remaining tested kinases (Src and ERK1/2) (Fig. 3a) as well as we did not see any activation of Akt in more aggressive cell lines HTB9 and HT1376 9 (Fig. 3b).

### Androgen mediates AR translocation

Androgen receptor usually locates in cytoplasm in complex with heat shock protein. Ligand-activated AR is known to translocate to the nucleus and subsequently activates transcription of several target genes. Cytoskeletal proteins facilitate nuclear targeting of AR. We checked intracellular localization of AR in the presence and absence of DHT. Western blot analysis of subcellular fraction (Fig. 4) shown that, without ligand, AR is located largely in the cytoplasm, but after incubation with DHT, AR is still present in cytoplasm but also undergoes preferentially to cytoskeletal fraction and membrane fraction and small amount are present in nucleus. These results suggest that DHT in bladder cancer activates not only the canonical AR signaling but also the non-genomic AR signaling.

### AR and Akt interact in lipid rafts in membrane fraction

Androgen receptor non-genomic signaling pathway can start as well at the plasma membrane. AR associates with plasma membrane lipid rafts and facilitates AR activation of these pathways. AR can localize to lipid rafts and interacts with and activate Akt independently of PI3K, which is usually required for downstream activation of Akt. Western blot analysis of subcellular fraction has shown visible increase of Akt level likewise AR protein in membrane fraction after androgen stimulation in comparison to control non-stimulated cells in both bladder cell lines HCV29 and T24 (Fig. 4).

### Androgen/AR signaling modulates translation initiation

**Fig. 5:**
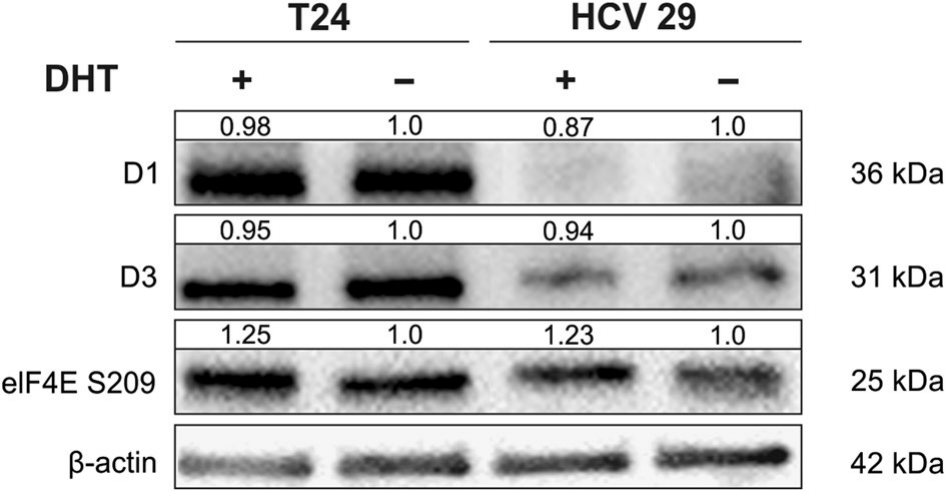
DHT treatment modulates translation by the cap-dependent mechanism. Densitometry was used to normalize to β-actin protein level and for quantitative comparison after DHT treatment

Cancer cells require elevated level of protein synthesis and translational control is critical for maintaining protein level. The majority of mRNA is translated by the cap-dependent mechanism. The increased level of free eukaryotic initiation factor 4E (eIF4E) and phosphorylated eIF4E can promote activation of cap-dependent translation. Housekeeping mRNAs require a low level of eIF4E, whereas proto-oncogenic and prosurvival proteins have a much higher dependency on phosphorylated eIF4E for translation. The increase of phosphorylated eIF4E promotes EMT and metastasis in cancer. We found that phosphorylation of eIF4E was increased after DHT stimulated androgen signaling, but we did not observe changes in cap-dependent translational control of cyclins D_1_ and D_3_ expression (Fig. 5).

### Effect of AR on EMT markers in bladder

**Fig. 6:**
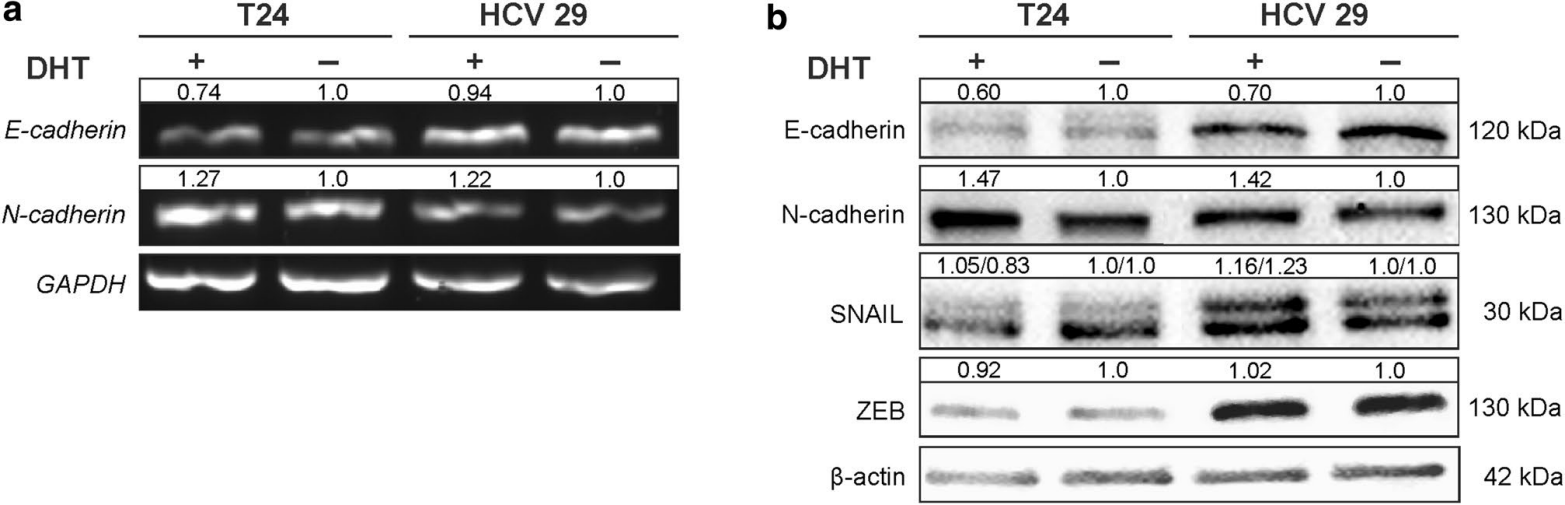
Effect of AR on EMT markers in transitional bladder cells. **a** E-cadherin, N-cadherin expressions on mRNA level were determined by reverse transcription-PCR 48 h after 10 nM DHT treatment. Densitometry was used to normalize to GAPDH level and for quantitative comparison after DHT treatment. **b** EMT markers on protein level. Densitometry was used to normalize to β-actin protein level and for quantitative comparison after DHT treatment. Representative results of at least three independent experiments are presented

Epithelial mesenchymal transition accompanies transformation from a quiescent cancer cells to a malignant phenotype. Loss of E-cadherin expression and induction of N-cadherin expression is a hallmark of the EMT process. Androgen-mediated AR signaling can induce EMT. Studied bladder cell lines: HCV29 and T24 express both E- and N-cadherins. HCV29 expresses comparable level of both E- and N-cadherins, but T24 expresses N-cadherin and a little E-cadherin at the protein and mRNA level (Fig. 6). We checked whether the AR signaling pathway might contribute to cadherin switch in bladder cell lines. As shown in Fig. 6, 48-h DHT stimulation was sufficient to downregulate the expression of E-cadherin in both cell types, and increase the expression of N-cadherin. As shown in Fig. 6 the expression of genes of a family of transcriptional factors including Snail and Zeb remains unchanged after androgen treatment, similarly like activity of metalloproteinases (data not shown).

### AR influences would healing

**Fig. 7:**
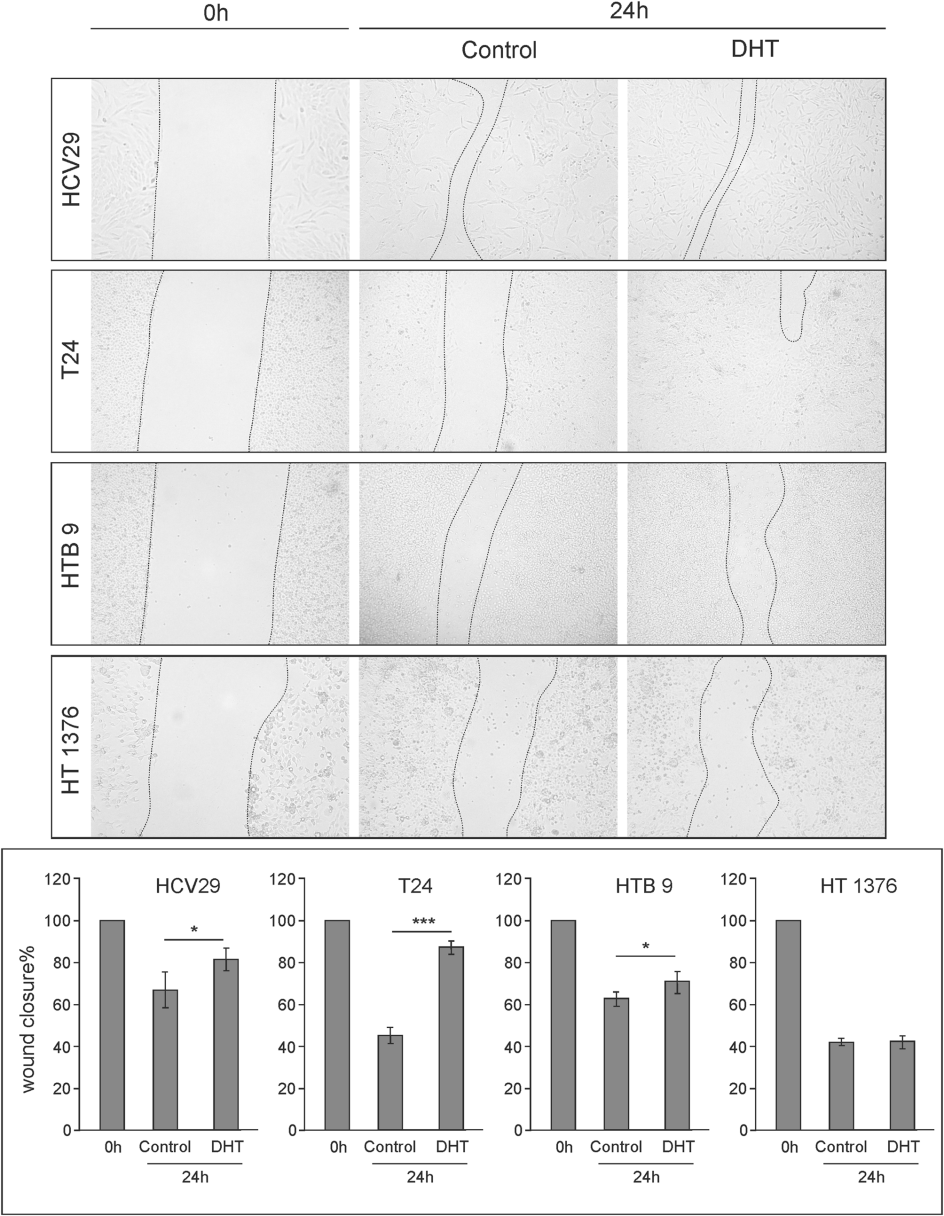
The effect of androgen (10 nM DHT) treatment on in vitro wound healing/migration assay. Data from at least four separate analyses were expressed as mean ± SD. Asterisks show significant differences between migration rate of control and DHT-treatment cells. Values are denoted as *p < 0.05 and ***p < 0.001

Cell migration and proliferation determine the rate of wound healing. Scratch wound healing assays were performed to evaluate the effects of DHT on bladder cells’ migration/proliferation. The results demonstrated that androgen treatment significantly accelerated wound closure after 24 h in transitional cell carcinoma T24 and in the transitional epithelial cells HCV29. We observed a slight supporting effect of DHT treatment on wound healing in HTB9 derived from men as more aggressive cell line, however the effect was invisible in HT1376 from grade III carcinoma, which is of female origin (Fig. 7).

## Discussion

Both genetic and environmental factors contribute to the initiation, progression and development of urinary bladder cancer (UBC). Using molecular classification in bladder cancer cell line models, we recognized that bladder cell lines showed differential expression of androgen receptor in male origins cell lines and responded differently to androgen stimulation. We found that nonmalignant transitional epithelial cells and transitional cell carcinoma had higher levels of AR expression compared to high-grade or muscle-invasive tumor cell lines. Also, immunohistochemistry study by Miyamoto et al. or Boorjian and colleagues indicated that down-regulated AR expression in UBC may be associated with tumor progression [22, 23]. However, other studies presented conflicting data [24]. Additionally, we observed very significant increase in androgen protein level after supplementation of DHT only in nonmalignant and carcinoma transitional cells. Androgens are known important regulators of AR mRNA and protein through transcriptional and post-transcriptional mechanisms. AR is ligand-dependent transcriptional factor that regulates gene expression, ligand binding profoundly increases AR stability and this stabilization is relatively unique to AR as other steroid receptors undergo hormone-mediated downregulation [25]. AR increases more than three times in the presence of androgen in prostate cell line LNCaP. The half-life of ligand-bound AR protein levels is also sensitive to regulation by cap-dependent mRNA translation [26]. Mikkonen et al. have shown that androgen administration significantly upregulated AR expression in murine lung and modified gene expression in a human lung cancer cell line [4]. Higher level of androgens in men than in women may be a reason for increased AR protein level and intensification of AR signaling especially at early stages of tumor initiation and progression of BC. After androgen stimulation in both cell lines HCV29 and T24, we observed AR translocation to the nucleus and also increased AR amount in cytoskeletal fraction. Interaction of AR with cytoskeletal proteins facilitates nuclear targeting of AR and probably increased of AR transcriptional activity which plays important role in G_1_/S phase transition. The cytoplasmic action of AR is observed also in non-prostate cells, such as fibroblast, where the level of AR is relatively low, and AR translocation in response to androgen is not observed [10]. However, non-genomic AR signaling also promotes cell proliferation and survival. Activation of AR signaling causes the regulation of numerous signaling pathways PI3K/Akt, Src and MAPK/ERK that in turn regulate cell proliferation. In addition, non-genomic effects of DHT can manifest independently, or in tandem, with the other genomic effects, to initiate androgen responses.

**Fig. 8:**
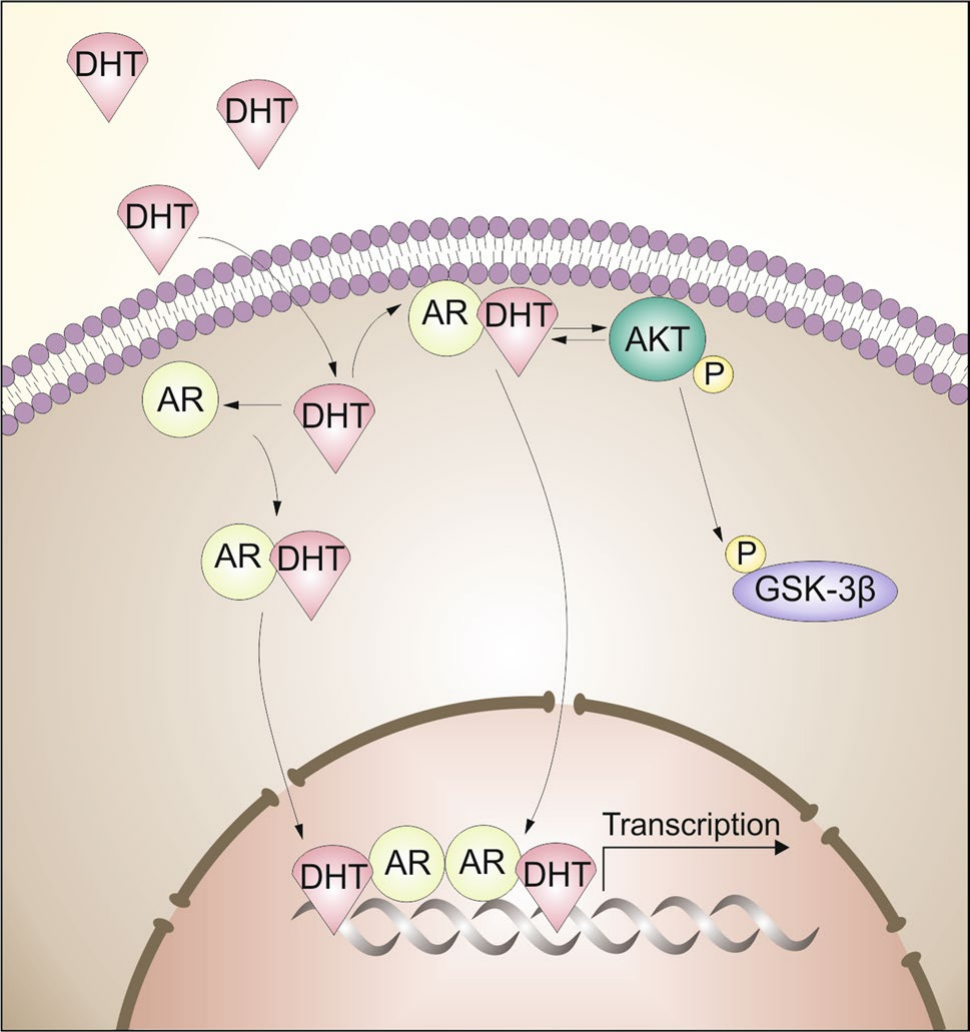
Model of the signaling pathway active in effect of DHT treatment in bladder cells. DHT interacts with AR, and translocates to nucleus to elicit transcriptional regulation of target gene. Note that DHT also stimulates activation of Akt kinase pathway. Akt kinase, after binding to plasma membrane is activated and stimulate AR/DHT complex to nucleus or/and regulate GSK-3β activity via its phosphorylation

We showed that androgen stimulation of bladder nonmalignant and cancer cells resulted in the activation of the PI3K/Akt pathway. Upon ligand binding AR interacts directly with the p85α regulatory subunit of PI3K, resulting in phosphorylation of Akt. We observed the increased phosphorylation of downstream signaling target protein kinases Akt and glycogen synthase kinase-3β (GSK-3β), whereas the levels of Akt and GSK-3β protein remained almost unchanged. Cinar et al. proved that AR upon binding DHT can activate Akt kinase in lipid rafts and mediates non-genomic signaling independent of PI3K [14]. Data suggest that AR associates with plasma membrane that facilitates activation of these pathways. The molecular mechanism controlling recruitment of AR to the plasma membrane is unclear. We also noticed the increase of AR level in plasma membrane fraction and this was accompanied by increase of Akt in this same fraction (Fig. 8). Interestingly, not all cell types that demonstrate a rapid androgen response express the classical nuclear AR. In the literature, the observation of non-genomic androgen-mediated activation of signaling pathways occur through different mechanisms in different cell types [27]. However, cytoplasmic AR signaling may also function through mitogen-activated protein kinase (MAPK) signaling, converging on extracellular signal regulated kinase (ERK) activation [28]. Another well-studied signaling molecule activated by steroid receptors is Src. In addition Src may also activate Erk 1/2 signaling cascade but we did not observe changes in phosphorylation level of Erk1/2 or any increase of Src kinase activation. Src activated by AR, can also stimulate proliferation and survival of prostate cells by enhanced PI3K signaling pathway. Non-genomic signals from AR to Akt may omit both PI3K and Src. Our data indicated that DHT-mediated AR-dependent Akt phosphorylation involved PI3K or is a result of direct interaction of Akt and AR in bladder cell lines. It is well known that Akt plays central role in cancer progression. This critical pathway acts as a convergence point for a multitude of upstream signals and in turn stimulates the activity of numerous downstream effectors, thereby mediating enhanced cellular survival, growth, protein synthesis, motility, and other functions of protumorigenic impact [29]. Accordingly, it is not surprising that deregulation and aberrant activation of the PI3K/Akt signaling pathway are common molecular events in a wide range of malignancies. Additionally, DHT-mediated activation of Akt is AR dependent in bladder cancer. Our results indicate that non-genomic signals involving AR in bladder cancer likely diverge at the level of cytoplasmic and plasma membranes. It is possible that AR non-genomic activity ultimately serves to influence AR genomic activity, because AR-activated kinase Akt can phosphorylate and activate AR in autocrine positive feedback loop [11]. Miyamoto and colleagues documented that AR involving genomic or non-genomic signals can also induce bladder tumor [30]. They showed that ARKO (AR knockout) mice do not get chemically induced bladder carcinogenesis, but when ARKO mice were supplemented with DHT, 25% of them developed bladder tumors. The ARKO mice were generated by a disruption of exon 2 of the AR gene, which encodes the AR DNA-binding domain. They provided the first in vivo evidence that AR in urothelium might play a crucial role in initiation and progression of urinary bladder cancers in genomic and non-genomic way. In addition AR inhibition attenuates tumor growth of T24 xenografts [31].

Activation of AR causes the regulation of numerous signaling pathways that in turn regulate EMT, which is early step in metastatic progression. EMT is a process by which epithelial cells lose their epithelial features and obtain a mesenchymal phenotype. Loss of E-cadherin expression and induction of N-cadherin expression is a hallmark of the EMT process [32]. A reduction or loss in expression of E-cadherin and the increase of N-cadherin expression in bladder cancer have been shown to be important in cancer progression [33]. Androgen-mediated AR signaling was also described to induce EMT in bladder cancer cells [34, 35]. Our data demonstrate that AR signaling can regulate the “cadherin switch” in bladder nonmalignant transitional epithelial cells and cancer cells. Robichaud et al. reported that EMT is regulated also by cap-dependent translation in mammary tumor model. They showed that eIF4E phosphorylation on Ser 209 is important for EMT and invasion [36]. It is also known that eIF4E activity is essential for controlling proliferation and invasion in bladder cancer [37]. Availability and phosphorylation of eIF4E are regulated by various upstream kinases and one of them is Akt. In our study, we found the slight increase of phosphorylation of eIF4E after DHT stimulation of androgen signaling. Considering how important factor is translational control in tumorigenesis, our result is not without significance.

It has been suggested that muscle-invasive bladder cancers are initially androgen sensitive for their growth but the sensitivity is lost during the progression due to activation of set of genes involved in metastasis. Activation of androgen/AR genomic and non-genomic signaling probably is not a driver in male bladder oncogenic transformation, but is a major player in progression of those cancers. Therefore, androgen-activated AR signaling is an attractive regulatory target for the inhibition or prevention of bladder cancer incidence in men.
